# Flexible work hours at the SUS: labor legislation at a specialized outpatient facility in Brazil

**DOI:** 10.47626/1679-4435-2024-1288

**Published:** 2025-01-31

**Authors:** Rodrigo França Gomes, Marco Antonio Pereira Querol

**Affiliations:** 1 Public Health Department, Faculdade de Saúde Pública da Universidade de São Paulo, São Paulo, SP, Brazil.

**Keywords:** legislation, labor, legislation, medical, physicians distribution, work hours, surveillance of the workers health, legislação trabalhista, legislação médica, distribuição de médicos, jornada de trabalho, vigilância em saúde do trabalhador

## Abstract

**Introduction:**

The geographical shortage and poor distribution of physicians, their traditional autonomy with multiple jobs, and low salaries they are paid in the public sector have created a reality of widespread failure to comply with the work hours defined in the Brazil Unified Health System legislation, causing legal uncertainty for managers and physicians.

**Objectives:**

This report aims to analyze the perception of managers, health personnel, and users of the Unified Health System about the corporate experience of a new labor legislation that preserves statutory labor rights and sets flexible work hours for medical specialists in Praia Grande, São Paulo, Brazil.

**Methods:**

This is a qualitative study with a phenomenological basis, using ethnographic research. A total of 42 social actors were interviewed in person or remotely with a digital recorder, including managers, physicians, and clients of the Unified Health System at a specialized outpatient facility (mean interview length 24.1 minutes [95%CI 17.7-30.6]), and official documentation.

**Corporate Experience:**

Emic and ethical perspectives from 489 units of meaning were compiled into 10 categories for analysis and presented in an ethnographic report for regrouping through nomothetic analysis, and records from an ideographic perspective for validity through integrative synthesis.

**Conclusions:**

The new legislation provided professional satisfaction, atracting and retaining specialist practitioners, providing legal certainty with external audit bodies and improving access to diagnostic and therapeutic procedures, microregulation between specialists and Primary Health Care, standing out as a successful experience for external audit bodies and atracting managerial interest from all over Brazil.

## Abstract (pt)

**Introdução:**

A escassez e a má distribuição geográfica de médicos, sua tradição de autonomia com múltiplos vínculos e os baixos salários praticados no setor público estabeleceram uma realidade de disseminada inobservância ao cumprimento da jornada de trabalho definida na legislação nacional do Sistema Único de Saúde, gerando insegurança jurídica a gestores e médicos.

**Objetivos:**

Analisar a percepção de gestores, trabalhadores da saúde e usuários do Sistema Único de Saúde sobre a experiência corporativa de uma nova legislação trabalhista que preserva direitos trabalhistas estatutários e estabelece uma jornada com horário flexível para médicos especialistas no município de Praia Grande, São Paulo.

**Métodos:**

Foi realizado um estudo qualitativo de base fenomenológica, por meio de pesquisa etnográfica. Usando gravador digital, foram entrevistados 42 atores sociais, de maneira presencial ou por videoconferência, incluindo gestores, médicos e clientes do Sistema Único de Saúde do ambulatório de especialidades da cidade (média de duração individual de 24,1 minutos [intervalo de confiança de 95% 17,7-30,6]), bem como documentação oficial.

**Experiência Corporativa:**

As perspectivas êmicas e éticas de 489 unidades de significados obtidas foram compiladas em 10 categoriais de análise e disponibilizadas em um relatório etnográfico para reagrupamento por análise nomotética, bem como registros sob perspectiva ideográfica, buscando validade por síntese integrativa.

**Conclusões:**

A nova legislação proporcionou satisfação aos profissionais, captação e fixação de médicos especialistas, conferindo segurança jurídica junto às instâncias de controle externo e aprimorando o acesso a procedimentos diagnósticos e terapêuticos, bem como a microrregulação entre especialistas e a Atenção Básica. Destacou-se como uma experiência exitosa por instâncias de controle externo e despertou o interesse por informações de gestores de todas as regiões do Brasil.

## INTRODUCTION

In accordance with articles 37 and 196 of the Brazilian Constitution^1^ and other subconstitutional legislation,^2,3^ the hegemonic form of public call notices for physicians in health services of the various states in Brazil binds the fulfillment of the work hours to the paradigmatic workload, which must be limited to a maximum of two public jobs with up to 60 hours per week, under penalty of the need to prove compatibility.^4^

This corporate experience report is set in Praia Grande, São Paulo, Brazil, a major municipality on São Paulo southern coast with 325,226 inhabitants, 100% Primary Health Care (PHC) coverage and 1.9 doctors per 1,000 inhabitants (compared to the national average of 2.6 doctors/1,000 inhabitants), according to the local 2022 *Relatório Anual de Gestão* (RG, Annual Management Report).^5^

In May 2015, the municipality passed Municipal Complementary Law No. 701/2015,^6,7^ which set forth medical work hours based on work output. The law basically converted 20 weekly work hours into 240 consultations per month (three patients per hour or 20 minutes per patient). It is based on voluntary agreement, maintenance of all statutory labor rights in force, increased remuneration linked to work output, and failure to comply with the law is punished with termination of the agreement and restoration of the obligation to biometrically record work hours on an hours-per-week basis.

In response to the recommendation of internal and external audit bodies to tackle failure to comply with medical work hours, this legislation is the result of a joint effort between managers and medical specialists to tackle a problem that is prevalent in Brazilian municipalities, i.e. the shortage and poor geographical distribution of physicians,^8,9^ autonomy and multiple jobs and interests as self-employed professionals,^10,11^ low salaries, precarious contracting arrangements, and high turnover in the public sector,^12,13^ which subject managers and health personnel to a domestic environment of legal uncertainty, informal agreements, and convictions for failure to comply with work hours by the State Court of Accounts.^14^

### OBJECTIVE

This report aims to analyze the perception of managers, health personnel, and users of the Unified Health System (SUS) about the corporate experience of a new labor legislation that preserves statutory labor rights and sets flexible work hours for medical specialists in Praia Grande, São Paulo, Brazil.

## METHODS

This is an analytical and exploratory study with a qualitative approach based on phenomenology^15^ and ethnographic research^16^ of documents and administrative processes that formed the historical social context, and semistructured interviews conducted at a medical specialties outpatient facility with 42 social actors involved in this corporate experience.^17^

Following consecutive non-random convenience sampling, 14 SUS users, 19 professionals from various medical specialties, and 9 managers were interviewed. The Informed Consent Form was signed in printed format or electronically, depending on the volunteer’s preference for face-to-face or online interview recorded on a digital recorder.

The digital recordings of the interviewees (E) were transcribed into units of meaning in a spreadsheet^18^ and confidentially identified by their social role and an identification unit containing the leter “E” followed by the time, in hours (h), minutes (min) and seconds (s) when the excerpt was taken from the interview. After confronting emic and ethical perspectives, they were compiled into 10 categories and included in an ethnographic report for regrouping through nomothetic analysis, and records from an ideographic perspective for validity through integrative synthesis.^19^

The study was authorized by the municipal scientific commitee and substantiated Opinion No. 4.015.170/CAAE: 26488619.8.0000.5421, dated May 8, 2020, and all documentation, including the ethnographic report used in the nomothetic analysis, is available at htps://drive.google.com/file/d/16-2KFzXGz8fO83UlqClApwHc9tzFXftz/view?usp=drive_link.

### CORPORATE EXPERIENCE

This documentary research of administrative processes in Praia Grande^6^ describes details of how Complementary Law 701/2015 replaced a weekly 20-hour work week with monthly output of 240 consultations. Regulated by a service order issued by the Head of the Health Department,^7^ the new legislation provides full autonomy for professionals to choose their work hours and the days of the week they work, provided that the availability of consulting offices is observed, under penalty of drawing or taking turns, and providing administrative and accounting details for the calculation of output.

The regulatory service order suggests, but does not oblige, that 312 consultations/month be scheduled to compensate for historical absenteeism, defining that patient or professional absences (sick leave) must be replaced in order to guarantee a minimum of 240 consultations/month (unless there are no patients waiting in the line, in which case the professional would be excused from atending the service).

The service order also recommends not exceeding four patients/hour and introduces a qualitative aspect called “Equivalence,” in which diagnostic and therapeutic procedures that involve a medical act, including outpatient surgeries, could be included in the specialists monthly consultation target through an arbitrated conversion parameter, broadening the diversity of specialized outpatient services offered.

As for the interviews, 1,012 minutes of digital recordings were collected from the 42 social actors interviewed, with a mean recording time of 24.1 minutes (95%CI 17.7-30.6).^18^ A total of 489 transcriptions of units of meaning were obtained and compiled into 10 categories, according to the Table 1, which describes the main subject of each category and provides figures for the contribution per social group.

**Table 1:** Organization of the 489 units of meaning into 10 categories

Categories	Units of meaning per social group			
	Managers	Medical specialists	SUS users	Total
01 - Compliance and impressions of the municipality law	20	23	5	48
02 - Remuneration and labor rights	14	25	0	39
03 - Attracting and retaining professionals	15	14	1	30
04 - Work hours and absenteeism	30	18	1	49
05 - Monitoring and external control	34	23	0	57
06 - Interfederative experiences	22	36	21	79
07 - Productivity and quality of care	32	17	25	74
08 - Production equivalence	12	13	0	25
09 - Health access regulation	11	20	0	31
10 - Suggestions for law improvement	9	48	0	57
Total	199	237	53	489

SUS = Sistema Único de Saúde (Unified Health System).

The 10 categories were compiled from the eight potential domains originally proposed in the semistructured interview model, with 199, 237 and 53 units of meaning coming from SUS managers, workforce, and users, respectively, into an ethnographic report with 214 selected units of meaning, according to the electronic address provided in the Methods.

From the statements that emerged in Category 01 – Adherence and impressions of the new municipal law, the interviews revealed that the specialist physicians fully complied with the law, emphasizing the assurance provided by the statute.

The creation of the SUS, through Federal Law No. 8,112/1990,^2,3^ defined the public competition with a 20-hour work week as the recommended hegemonic form of hiring in the SUS, which was extended to the other states through the principle of hierarchy of norms.

However, the historical existence of public and private partnerships in the National System^20,21^ and a poorly distributed shortage of physicians^11^ have led to a reality that is contrary to what was idealized, with the creation of multiple precarious employment relationships, low pay, long distances between home and the various workplaces, high turnover and failure to comply with the workload, to the detriment of the quality of work in the SUS.^8,12,22,23^

In Category 02, remuneration and labor rights, the testimonials converge in criticism of the low pay in the public sector. The price of a medical consultation in specialized care has been frozen since 2008 at R$ 10.00,^24^ and the responsibility for effectively paying for specialist physicians falls on municipalities, which, when they are unable to provide them – either due to a shortage of specialists^11^ or budget limitations – struggle with access restrictions and overload state and regional reference facilities for this possible guarantee, resulting in long waiting lines in most Brazilian municipalities.

The interviewees reported that the municipal law offered atractive pay (approximately five times the SUS reference rate in its highest range) and guaranteed legal security not found in other public jobs where informal agreements predominate, as it officially relaxed working hours and protected all statutory labor rights.

Thus, this municipal law addresses the contradiction between the norm and the reality created on the domestic scene, confronting the legal uncertainty experienced by managers and workers. According to Echtemacht,^25^ occupational illness can be the result of adverse realities constructed from a complex interaction between the concreteness of the human condition and the atribution of meanings. The author warns that social determinisms may not act directly on biological order, but through consciousness, sociality and instrumentality, which are both set and interact as mediators of human activity as a psychobiological construction, which would imply health surveillance of these professionals on a nationwide scale and the search for solutions that deal with the contradictions established in a broad social and historical context.

In Category 03, which deals with atracting and retaining professionals, it was revealed that the municipality has managed to atract specialists as a result of the law, even correcting problems of chronic shortage of supply, such as in neuropediatrics, hematology, pulmonology, oncology, child psychiatry, and other other subspecialties, which are usually only recruited as legal entities in large cities.

In terms of work hours and absenteeism in Category 04, the conflict over registering work hours has been resolved, with an end to the historic threats of resignations. Managers also report a decline in medical absenteeism. Despite the law ensuring full pay for legal reasons, such as sick leave, the need to cover the minimum monthly output and the prospect of higher salaries for output inhibit absenteeism, but not without criticism (“*sick leave, you got sick, that’s your business. You have to go another day to make up for the day you missed. So, I don’t even issue sick leaves anymore. I go whenever I can*” - dermatology, E26). There is also a greater commitment to patients to reschedule appointments.

Category 04 was praised for ensuring more flexible work hours, which allowed physicians to maintain other public jobs and private practices. Among the criticisms was the management leniency in working only one day a week (which has benefited physicians who live in other states, but at the expense of care and bureaucratic flows), potential presenteeism and pressure for offices on specific days of the week. Thus, like the criticisms pointed out by Costa et al.^26^ about the 2017 Labor Reform – which potentially affected breaks for rest, relaxation, and meals during the working day – the flexibility of this new municipal law creates potential space for occupational risks.

Category 05 emerges from reports on the monitoring and action of external audit bodies, which include the triad of 1) findings of failure to comply with work hours, 2) discourses of collective dismissal, and 3) adoption of mandatory measures.

In a survey of 20-year history of the public digital collection of decisions by the State Courts of Accounts across the country, in their role of external control,^6^ monitoring the provision and fulfillment of the weekly workload of physicians in municipalities, Gomes & Querol^14^ reported that the decisions handed down frequently include compulsory measures (67.6%) against public managers and the medical practitioners involved, consisting of fines, compensation, and the compulsory dismissal of public workers.

Once the municipal law was implemented in 2015, despite initial resistance from these bodies, particularly from the point of view of concerns about the lower quality of consultations and services, and suggestions for improving monthly output (from 240 to 320 consultations/month, compensating for average absenteeism), this was overcome and the municipality was cited as a potential model for tackling the problem.

Similarly, Category 06, which compiles interfederative experiences, reinforces the report of legal insecurity health practitioners experience in other municipalities that maintain informal mechanisms to address failure to comply with work hours, and the spread of the new law among health practitioners and the control bodies themselves, including visits by health managers from various regions of Brazil for benchmarking and exchanging experiences.

Category 07 discussed the relationship between work output and quality of care. Users complained about the waiting time for appointments with specialists, though no direct criticism was leveled at the quality or length of medical consultations. As for doctors and managers, their perceptions differ. Some point to a loss in time and quality of care and others that there have been no changes in the way care is provided under the law, which is dependent on the physician’s profile. There were reports of professionals who were not working the required daily hours and who started working more than 20 hours a week, and some even applied for a second public competition for another contract, lured by the higher pay.

The managers reported an increase in work hours and pressure for offices with the passing of the law, which establishes an output parameter of 80 consultations per week with an estimated consultation time of 20 minutes per patient, and 40 minutes in the Psychosocial Care, Maternal and Child Care, and People with Disabilities. However, there are reports of physicians who only see patients once a week, providing a consultation on average every 6 minutes. These professionals complain about the self-imposed excessive workload on a single day of the week. Classic studies from the 20th century report medical consultation time of 5 minutes on average, increasing to 7 minutes and 46 seconds with the advent of computerization.^27^

A systematic review conducted in 2017^28^ examined 28 million consultations in 67 countries and found that consultations of 5 minutes or less were detrimental to quality. The municipality choice of imposing a minimum allowed time of 7.5 minutes per consultation in the legal text establishes a pragmatic view of reality, while the option of seting an output parameter of 20 or 40 minutes per consultation documents the intention to offer physicians conditions for quality consultations.

Classified as output equivalence, Category 08 reports on the new legislation strategy of valuing the offer of diagnostic and therapeutic procedures that involve the medical practice. Examples include procedures for collecting blood gases, cerebrospinal fluid, ultrasound, electroneuromyography, and outpatient surgical procedures. These procedures were often a source of conflict between doctors over which specialty would be responsible for the care provided. The interviews show the expansion of the outpatient services portfolio, shorter waiting lists for specific procedures and the pursuit of professional development by specialists motivated to offer other services, not only resolving the conflict between specialties but also generating competition between professionals and access to procedures that were once scarce.

In terms of improving access to health care in the SUS, Bertussi et al.^29^ point out that deconstruction also involves specialized care, identifying professionals who are willing to act under a different logic, in different places and, above all, to participate in the collective construction of care in a shared network. The municipal law has produced results that go beyond standardized improvements, engaging many specialists in processes of microregulation and continuing education in their specialties.

As examples of improvements in the regulation of health access, Category 09 reports suggest an increase in the supply of consultations and a decrease in demand. In some specialties, professionals began to atend PHC continuing education courses, build access protocols, make counter-referrals and actively participate in regulation, seeking to reduce waiting lists and their monthly workload dedicated to the municipality in person. Thus, voluntarily contributing to the regulatory functions of control, balance, adaptation, and direction.^21,30^ However, this experience was not homogeneous between specialties and professional profiles, with some focusing on increasing output and apathetic to interaction with PHC.

Finally, in terms of suggestions for improving the municipal law, in Category 10, the interviewees suggested procedures to be included as equivalents, bonuses for enrollment and teaching for PHC, other qualitative mechanisms for measuring output and balancing differences between specialties, requiring the use of electronic medical records for adherents and creating proportionality of output according to the number of business days per month, allowing for less output in months with many holidays and for official continuing education activities (such as leave for conferences and university extensions).

## CONCLUSIONS

In an environment of resumption of non-covid-19 care, with a probable worsening of the waiting lists for medical specialties and increased activities of external oversight on the fulfillment of the work hours of these professionals in the Brazilian public sector, this ethnographic research analyzes a corporate experience of implementing a municipal law that has been presented as a proposed solution to this problem, providing legal certainty, retaining medical professionals, and improving access to medical care, while potentially generating presenteeism and compromising the quality of medical care.

In an atempt to broaden the academic and interfederative dialogue on the fulfillment of the work hours of physicians in the SUS, we suggest that future studies be conducted, reporting on other successful experiences in the various regions in Brazil, and seeking to understand the context and functioning through an appropriate theoretical approach.
